# Following the central dogma: Nanopore sequencing from DNA and RNA to proteins

**DOI:** 10.1016/j.isci.2026.114835

**Published:** 2026-01-29

**Authors:** Yutong Wang, Qian Han, Yilong He, Shumin Deng, Ziwei Huang, Ziyan Zhang, Shuaijian Dai, Zhuxin Dong

**Affiliations:** 1 Department of Biomedical Engineering, School of Basic Medical Sciences, Central South University, Changsha, Hunan 410013, China; 2 Furong Laboratory, Changsha, Hunan 410000, China; 3 National Engineering Research Center of Personalized Diagnostic and Therapeutic, Changsha, Hunan 410000, China

**Keywords:** Techniques in genetics, Sequence analysis, Methodology in biological sciences

## Abstract

The first nanopore-based sequencer was launched in 2014, and subsequently, nanopore played an irreplaceable role in disclosing the first complete, gapless sequence of a human genome in 2022 due to its megabase-scale read lengths. However, a striking revelation from DNA sequencing is that over 95% of human DNA does not specify a protein, which means tremendous proteomic information cannot be predicted from the genome. Therefore, nanopore researchers have been leaning increasing attention to the proteome. Nowadays, nanopores have demonstrated unprecedented performance in discriminating individual proteinogenic amino acids with chemical modifications. Meanwhile, diverse strategies for full-length proteins to translocate through nanopores have been developed. Undoubtedly, nanopore will sooner or later facilitate *de novo* protein sequencing. This nanopore review begins with DNA sequencing and elaborates on up-to-date technical breakthroughs in protein sequencing and other proteomics approaches. Overall, nanopore technology is conducive to discovering the proteome diversity and revealing the pathogenesis mechanism.

## Graphical abstract

**Figure:**
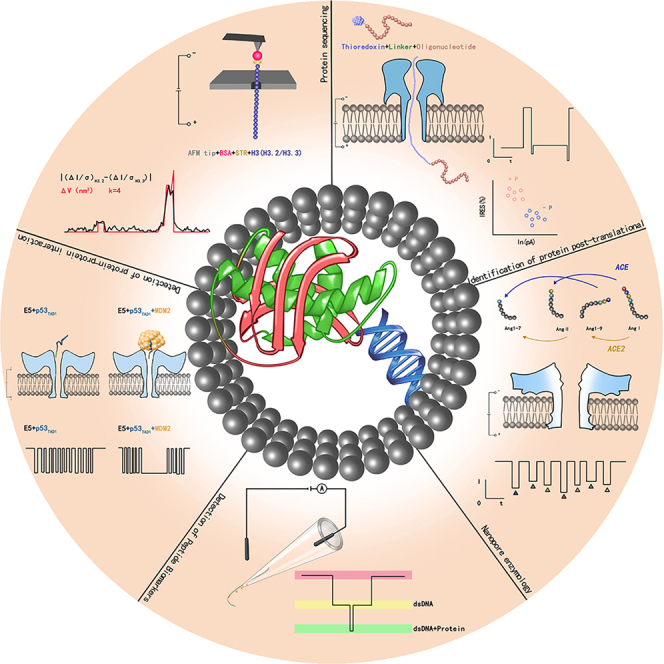

## The emerging nanopore technology

DNA is the main genetic material of most creatures. It stores the information that guides almost every biological activity. Since Watson and Crick published the structure of DNA in the 1950s, the curiosity about learning DNA has never stopped.^1^^,^^2^ With the progressive discovery of the double-helical structure and the Chargaff Law, scientists have been striving to unravel all mysteries of DNA,^3^^,^^4^^,^^5^ among which DNA sequencing is admired.^6^^,^^7^ In 1977, Sanger initiated a DNA sequencing technology called the Chain-Termination Method or Dideoxy Method to determine the full nucleotide sequence in phage f1 and ФX174 DNA.^8^^,^^9^ Then, Maxam proposed the Chemical Degradation Method, which together with Sanger’s is known as the first-generation sequencing technology, “sequencing by synthesis.”^10^ Through modifications by connecting fluorescent groups to oligonucleotide primers or ddNTPs to form a more rapid and automatic DNA sequencing approach,^11^^,^^12^ or using fluorescent primer to replace radio labeling,^13^ the advanced Sanger’s method adopted capillary electrophoresis on the basis of the chain-termination method and facilitated high throughput sequencing during the 1980s. Based on capillary electrophoresis, researchers launched the first semi-automatic DNA sequencer in the 1990s,^14^^,^^15^ which is still widely used today. Toward *de novo* sequencing, the shotgun DNA sequencing proposed by Anderson et al. showed the characteristics of high speed with simplicity and low cost, though, lack of accuracy, which was then employed in the Human Genome Project sponsored by the International Human Genome Sequencing Consortium (IHGSC) and Celera Genomics in 2001.^16^^,^^17^ Based on the draft sequence of the euchromatic portion of the human genome uncovered in 2001, IHGSC adopted an advanced version of Sanger’s sequencing and accomplished the first euchromatic sequence of the human genome in 2004.^18^

The first-generation sequencing technology had a limited read length of 500–800 bp with an excellent accuracy of 99.9%, but it was inadequate for large-scale application owing to low throughput and high cost, even after automation. To overcome these constraints, the second-generation sequencing technology emerged, exemplified by platforms such as Illumina’s HiSeq/MiSeq/Solexa, Roche’s 454, and ABI’s SOLiD.^19^ The second-generation sequencing still operates on the principle of base discrimination using fluorescent signals (error rate ∼0.1%), but has a significant improvement in throughput enabled by the parallel analysis of tens of thousands of DNA fragments. However, the read length is unwillingly limited to 150-300 bp, which inevitably causes errors when assembling the overlaps into one long contiguous sequence read.^20^

The sequencing technologies discussed above have a characteristic in common: all require molecular amplification that would lose the information on base modification and take the risk of introducing artificial error and statistical bias. Fortunately, the third-generation sequencing represented by single-molecule real-time sequencing (SMRT) (PacBio) is based on single-molecule sequencing technology,^21^ and therefore amplification is no longer necessary.^22^ Although SMRT is another version of sequencing by synthesis, it possesses an impressive read length: its average read length reaches 15,000 bases, and can sometimes exceed 100,000 bases. Briefly speaking, SMRT uses specific fluorescent tags to label four bases, and identifies each added base according to the color of light flash from the tag under control of a single polymerase that is attached to the bottom of each zero-mode waveguides pit for *in vitro* synthesis. Besides sequencing, SMRT can detect chemical modifications by distinguishing the sequencing time interval between two adjacent bases. For example, a methylated base would slow down when passing through the polymerase, resulting in a long interval between two adjacent flashes observed.^23^ Even though the raw reads from SMRT fast sequencing exhibit a high error rate in single read (13–15%), they can lead to extremely accurate consensuses (>99%) that are built up rapidly by repetitive coverage of the same molecule.^24^

**Figure 1:**
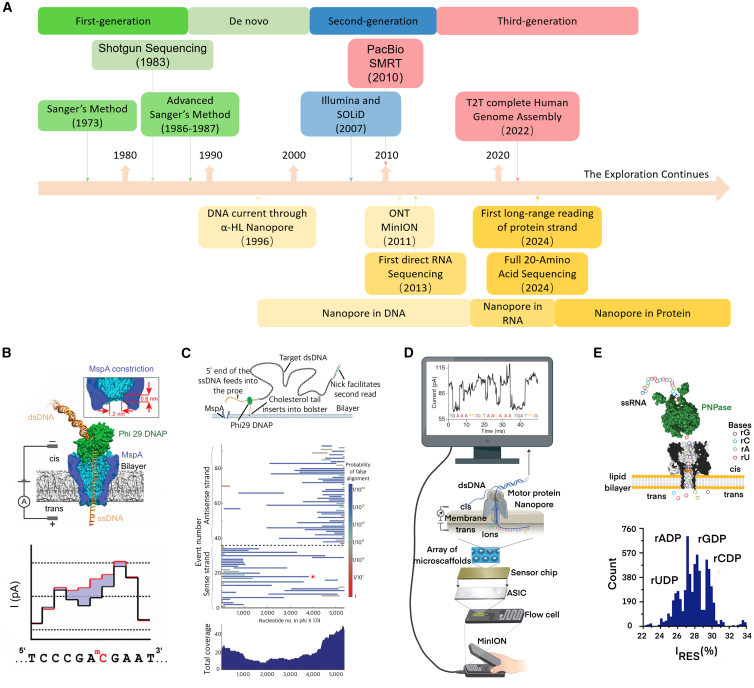
Nanopore DNA/RNA sequencing (A) The historical timeline of sequencing technology development and the parallel progress of nanopore sequencing. (B) Researchers conducted mutant MspA nanopore for single-molecule DNA sequencing with the help of phi29 DNA polymerase. The current trace showed the potential of this nanopore method in methylation detection. Reproduced with permission from ref.^28^ (C) Researchers achieved breakthrough improvements in both resolution and read length for DNA sequencing using engineered MspA nanopores, combined with DNA polymerase. The figure demonstrates the high-accuracy sequence prediction on Phi X 174 genome up to 4,500 bases in length. Reproduced with permission from ref.^29^ (D) The MinION device (Oxford Nanopore Technologies) performs scalable, real-time DNA sequencing using biological nanopores. Reproduced with permission from ref.^30^ (E) The application of biological nanopore technology in RNA sequencing, in which nucleotides were cleaved from ssRNA to translocate through the nanopore. Reproduced with permission from ref.^31^

In 1996, Deamer et al. used α-hemolysin nanopore (α-HL) to detect transient current blockades caused by the passage of DNA and RNA, ushering in a new era of single-molecule sequencing technology.^25^ Nanopore sequencing differs from other technologies in that it depends on electrical signals instead of optical signals. The technique works by attaching two electrodes to electrolyte-filled *cis*- and *trans*-sides separated by a lipid bilayer into which transmembrane proteins can insert to form a nanoscale channel. Then, under the net effect of electroosmotic force (EOF) and electrophoretic force (EPF) due to a cross-membrane electric potential bias, DNA single molecules translocate through the nanopore, and each generates a characteristic current blockade that can be captured by a patch clamp amplifier and analyzed using computational tools.^26^ As a rising star, nanopore technology has been widely used for DNA/RNA sequencing, protein sequencing, and detection of biomarkers as well as metal ions by virtue of theoretical simplicity, low-cost, label-free, amplification-free, high-throughput, long read length, and real-time sequencing. In 2014, ONT launched a portable nanopore microarray-integrated microfluidic device for DNA sequencing, named MinION, making nanopore sequencing technology commercially available for the first time.^27^ Figure 1A summarizes the development timeline of sequencing technologies, including the progression of nanopore sequencing from DNA and RNA to protein.

## Nanopore DNA sequencing

### Optimization of nanopore

At present, nanopore techniques can be divided into two main types, categorized by material: the solid-state nanopore and the biological nanopore. The α-HL nanopore, that firstly demonstrated to have potential for DNA sequencing in 1987, belongs to the latter.^32^ α-HL monomer is water-soluble and can self-assemble into a heptameric nanopore with a 5-nm-long stem that penetrates through cell membranes or synthetic lipid bilayers.^33^ After more transmembrane proteins were discovered, researchers managed to select appropriate biological nanopores and tailor them to meet the needs of DNA sequencing. In 2006, the cyclodextrin adapter was equipped on the α-HL nanopore to first observe varying levels in current blockades associated with nucleotides passing in sequence, and then four oligonucleotide homopolymers were distinguished by the E111N/K147N modified nanopore.^34^^,^^35^ *Mycobacterium smegmatis* porin A (MspA) is another popular biological nanopore for DNA sequencing. Not only does MspA have strong chemical and temperature stability, but it also has a conical structure with a diameter of about 1.5 nm at the constriction, making it an excellent candidate to allow passage of ssDNAs.^36^ Gundlach’s team constructed M1MspA, M2MspA, and M3MspA mutants on the basic structure of MspA protein through site-specific mutation technology to solve the frequent spontaneous gating effect of WT-MspA, which laid the foundation for nanopore DNA sequencing.^37^ Subsequently, they realized long read length up to 4,500 bases of ssDNA by using MspA mutant nanopores and demonstrated the capability of post-translational modification (PTM) detection, such as 5-methylcytosine DNAs (Figures 1B and 1C).^28^^,^^29^^,^^38^ So far, researchers have also begun to explore the potential of other modified biological pores, such as Phi29 and Aerolysin (AeL) mutants, to improve sensitivity and develop polynucleotide detection.^39^^,^^40^^,^^41^^,^^42^ It is worth noting that artificial intelligence approaches, e.g., hidden Markov model and recurrent neural network (RNN), have become competent for base calling. For instance, a mutant of a bacterial amyloid secretion channel, CsgG, together with a helicase motor enzyme, composed the main mechanism in the MinION sequencer and reached ∼15 kb read length using the RRN base calling algorithm.^43^ Through continuous improvements in biological nanopore design and motor enzyme processivity, nanopore sequencing enabled ultra-long reads (>100 kb) that can directly span complex and repetitive genomic regions.^44^ Furthermore, the ability to detect DNA modifications at single-molecule resolution expanded the nanopore’s utilization in genome research.^29^

The development of nanotechnology has promoted the emergence of solid-state nanopores.^45^ Compared with biological nanopores, the geometry of solid-state nanopores can be adapted to various analytes. By narrowing the apertures for a single strand to pass through, not only can solid-state nanopores filter dsDNA and folded ssDNA molecules, but also slow down the translocation speed by increasing interaction between DNA molecules and the nanopore wall.^46^^,^^47^ In 2001, Li et al. were the first to try fabricating a single nanoscale pore on a silicon nitride membrane using ion-beam sculpting, and finally succeeded in reducing pore size to 1.8 nm after Ar^+^ beam exposure.^48^ Subsequently, researchers successfully fabricated 2-nm-diameter SiO_2_ and Al_2_O_3_ nanopores.^45^^,^^49^ To date, a number of methods have been developed to fabricate solid-state nanopores, including focused electron beam (FEB), focused ion beam (FIB), controlled breakdown (CBD), electrochemical reactions (ECRs), laser ablation method, and laser-assisted controlled decomposition.^26^^,^^50^ Dekker et al. employed a scanning transmission electron microscope (STEM) to facilitate the electron beam sputtering scheme, where the shape and size of nanopores were under precise control.^51^^,^^52^ Since then, e-beam sputtering has been one of the most common tools for solid-state nanopore fabrication. In 2003, Li et al. reported their study of using a solid-state nanopore device to detect ssDNA translocation and the DNA folding behavior, which marked the first DNA sequencing experiment using a solid-state nanopore.^53^ In 2004, Chang et al. detected dsDNA translocation signals through a nanopore in SiO_2_ membrane for the first time.^54^ To improve the resolution, 2D materials, such as graphene, were found capable of providing an ideal thickness for accommodating a single base.^55^ In 2010, Schneider et al. first managed to use a graphene nanopore for DNA detection.^56^ In order to solve the sticky interfacing of graphene materials to DNA molecules, single-layer MoS_2_ nanopores were proposed. The layer thickness of MoS_2_ is about 6–10 Å, equivalent to the length of two consecutive nucleotides.^57^ In 2014, Farimani et al. attempted to detect the current signals of dsDNA when translocating through a MoS_2_ nanopore. It was found that the signal-to-noise ratio (SNR) was improved to 15.02, and the MoS_2_ nanopore presented better resolution than the graphene nanopore.^58^^,^^59^ The SiN_x_, graphene, and phosphorene nanopores showed advantages in chemical and thermal stability, and thus have gone mainstream.^60^^,^^61^ In 2013, Venta et al. produced a 5-nm-thick SiN_x_ nanopore and successfully distinguished three homopolymers of ssDNA: poly (dA), poly (dC), and poly (dT).^51^ Besides membrane thickness, researchers have been exploring other schemes for improvement. For example, partial heating of a solid-state nanopore would induce DNA molecules to extend and be confined to the pore, which affects the electrophoretic force and slows down their translocation.^62^ Addition attachment, such as constructing dielectric coating inside solid-state nanopores, could form electrostatic traps and trigger base-by-base ratcheting of ssDNA.^63^ Tsutusi et al. provided an interesting approach by embedding electrodes on a SiO_2_ membrane to create a transverse tunneling current through a 15-nm-diameter nanopore and distinguished the bases in DNA oligomers. It was demonstrated that such a device could unambiguously distinguish GMP and TMP out of the nucleotide mixture.^64^ Glass nanopipette forms a special nanopore sensing system, whose diameter can generally reach tens to hundreds of nanometers. Although nanopipette lacks sensitivity on small molecules identification, it demonstrates some unique advantages over solid-state and biological nanopores, e.g., chemical and thermal stability, flexible geometry with excellent surface-to-pore volume ratio for convenient functionalization, repeatable and low-cost fabrication scheme. Liu et al. proposed a system combining a nanopipette with DNA carriers, which yielded high-affinity binding aptamers to α-Synuclein oligomers and eliminated the α-Synuclein monomers in the cerebrospinal fluid, so that patients with Parkinson’s disease were successfully differentiated from healthy controls.^65^

### Other optimization strategies

Under a transmembrane voltage bias, the typical residence time of DNA in a nanopore is less than 1 μs/nt, resulting in picoampere-scale variations in ionic current and insufficient resolution to distinguish different bases. By slowing down the translocation of DNA (>100 μs/nt), the SNR can be improved to meet the sequencing requirement.^66^ In addition to directly modifying nanopores, the translocation speed can be optimized by modifying translocating molecules or adjusting environmental parameters, so as to improve data acquisition and enhance nanopore resolution.^67^ In 2001, Vercoutere et al. first increased the average residence time by adding hairpin structures to both 3′ and 5′ ends of DNA. It was found that the more deoxynucleotides folded in the hairpin, the slower the translocation rate.^68^ Later, Manrao et al. innovatively coupled Phi29 DNA polymerase with α-HL nanopores, showing that polymerase was able to pull the captured DNA strand out of the pore against the electrophoretic force.^69^^,^^70^ Due to its exonuclease activity and strong affinity for DNA substrates, Phi29 DNA polymerase cleaved the 3′ end of DNA and directed double-stranded DNA synthesis, achieving a ratcheting motion at a specific rate ranging from 25,000 to 400,000 μs/nt.^71^ In 2014 and 2015, helicase, Dda in particular, was found to be useful for nanopore DNA sequencing.^72^^,^^73^ Unlike polymerases, which pull ssDNA out of nanopores against the electric field force, helicases mediate the translocation to sequencing-comfortable rates (e.g., >2,000 μs/nt) by braking the electrophoretic motion. Notably, a stalling region that prevents the inchworm-like motor progression until nanopore capture occurs is critical for long read length in ATP-containing electrolyte. In 2016, Church et al. designed specific oligonucleotide-based tags. The tagged complementary nucleotide was captured by the nanopore when the polymerase-mediated synthesis began, and generated a current blockade with signatures of each tag, indirectly identifying the base sequence.^74^ The translocation ambient conditions also impact the nanopore sequencing performance. In 2005, Fologea et al. managed to reduce the molecular translocation rate by at least 10-folds after adjusting the viscosity of electrolyte solvent, electrical bias voltage, salt concentration, and the ambient temperature, which promoted the base detection sensitivity.^75^ In 2010, the team of Dekker replaced the KCl solution with the LiCl solution and slowed down DNA translocation through the nanopore by 10 times.^76^ Later, Goto et al. used an alkaline CsCl aqueous solution to achieve the distinction of four DNA homopolymers by disrupting the hydrogen-bonding network between guanines.^77^ Furthermore, molecular dynamics simulation was first involved in nanopore sequencing in 2004.^78^ It was employed to carry out computational experiments to sequence ssDNA constituted by four different bases (A, C, G, T) along with 5-methylcytosine (mC) using a graphene nanopore with an aperture less than 1 nm. Such detection could reach a single-base resolution with the aid of atomic force microscopy (AFM).^79^

Furthermore, to explore beyond linear DNA sequencing, nanopores have been ingeniously applied to decipher the epigenetic and higher-order structural information of genome organization, such as chromatin complexes. A collaboration led by ONT developed an end-to-end workflow that combined chromatin conformation capture (3C) with nanopore sequencing, called Pore-C. Supported by nanopore, long-range, multi-way contact information can be obtained. Pore-C can directly sequence ligated DNA concatemers proximally, unambiguously identify multi-locus interactions, offer a genome-scale view of the 3D interactome, and detect native epigenetic modifications.^80^

### Advantages and limitations of nanopore DNA sequencing

Compared to traditional DNA sequencing technologies, nanopore takes into account the advantages of without amplification or labeling, long read-length, and high-throughput, and so forth. Specifically, solid-state nanopores offer desirable flexibility in pore size and film thickness, which will meet the needs of diverse applications. Besides, their robust mechanical, thermal, and chemical stability holds the potential for constructing large-scale, long lifetime biochips.^81^ However, solid-state nanopores generally lack specificity in distinguishing between distinct molecules with similar sizes.^82^^,^^83^ In addition, solid-state nanopores exhibit two main noise sources: the high-frequency noise from the material parasitic capacitance (capacitance noise) and the low-frequency noise due to the drift in the mean ionic current fluctuations (1/f noise).^84^ Despite the noise inference, to precisely fabricate massive solid-state nanopores with identical geometries is still challenging, which also leads to higher error rates. Unlike the smooth protein interior of biological pores, the surface of solid-state pores is more prone to non-specific interactions with DNA/RNA, which causes unstable translocation speeds.^52^

In contrast, biological nanopores can be reproduced at lower cost, and their self-assembly structure allows for the creation of multiplexed sensor arrays. The successful commercialization of MinION confirmed such an advantage of biological nanopores for high-throughput sequencing and adequate pore loss (<20% of viable pores every 8 h).^85^ Moreover, biological nanopores can be specifically engineered. For example, adaptors can be a powerful addition to nanopores through biochemical reactions.^86^ The primary error sources in biological nanopores stem from the intricate sequence context effect within the sensing region and the kinetic variability of the motor enzyme, which complicate signal decoding and cause timing inaccuracies. Additionally, unlike solid-state nanopores, biological pores are often vulnerable to extreme solution conditions, such as acidic/basic pH, low/high temperatures, low/high salt concentrations, and the presence of certain chemicals such as surfactant. Structural variations of the pore protein and the inherent fragility of the lipid bilayer further introduce signal instability and limit read length, compromising the overall accuracy.^87^ Due to the inherent characteristics of both the pore size and characteristics, biological nanopores are easily obstructed, which is particularly pronounced when detecting complex biological samples such as serum. Collectively, these factors severely restrict operational lifetime and user experience.^88^ Initially, the MinION platform exhibited an average read length of 30–100 kb limited by the enzymatic processivity. Since then, significant progress has been made, and the platform is now capable of producing megabase-scale reads. Additionally, while the upfront instrument cost is affordable, its proprietary reagents remain costly.^89^ It would be interesting to compare SMRT from PacBio with MinION from ONT. While SMRT offer superior base-level accuracy in long sequences ideal for variant discovery and high-confidence genome finishing, MinION is a straightforward sequencing approach with ultra-long read lengths and a popular portability as sketched in Figure 1D.^30^ On the one hand, the base calling error of a single read from SMRT is about 13% for average reads of 15,000 bases, and the error of the consensus with 50-fold coverage can be prominently reduced, yielding an accuracy >99%. On the other hand, even though MinION generally provides a lower accuracy (>90% for a single read), it can be improved by base-calling algorithm upgrade and pair reading (>97%) when directly sequencing DNA stands that are 1,000,000 bases long. Therefore, a combination of the two multiplexing high-throughput methods can be a more powerful DNA sequencing strategy.

## Nanopore RNA sequencing

DNA sequencing reveals the most important genetic information of human beings, enabling a fundamental understanding of the underlying disease mechanisms. However, DNA does not encompass all genetic information governing human physiological activities. RNA sequencing is indispensable for comprehensively understanding cellular and organismal functions.^90^ Crucially, the ability to directly sequence native RNA strands and simultaneously identify epitranscriptomic modifications within a single assay represents a huge need—and this is yet another paramount advantage of nanopore technology. In 2008, sequencing RNA from a mouse was first accomplished.^91^ Yet, due to the complexity of the human genome and the defects of traditional RNA-seq, accurately inferring transcriptome structure and relative abundance remains challenging. Meanwhile, a large number of mRNA molecules are required to generate sufficient cDNA for conventional RNA sequencing. Unfortunately, transcribing RNA into cDNA inevitably introduces multiple biases, incomplete transcript sequences, and random artifacts, all of which could mislead the characterization and quantification of transcripts and therefore jeopardize the RNA sequencing outcomes.^92^ In this regard, nanopore sequencing has emerged as a competent direct RNA-seq scheme, even though currently the motor protein (e.g., M1) ratchets RNA strand through a nanopore slower (70 nt/s) than DNA sequencing. Encouragingly, recent advances included the identification of nucleoside monophosphates and their epigenetic modifications of RNA, which was considered a milestone for nanopore RNA sequencing.^90^ In 2013, Bayley et al. first reported nanopore-based direct RNA sequencing. They broke down the phosphate groups of ribonucleotides using α-HL nanopores containing cyclodextrin adapters, and further improved the accuracy by adding guanidino-modified adapters (Figure 1E).^31^ Subsequently, Parker et al. used CsgG nanopore to detect the mRNA methylation (m6A) in 2020,^93^ and nanopore technology has been proven to be promising for recognizing N6-methyladenosine, N6, 2′-*O*-dimethyladenosine in RNA.^94^ An up-to-date breakthrough demonstrated that an engineered MspA nanopore could identify nucleoside monophosphates of RNA and their epigenetic modifications.^95^ Li et al. presented a nanopipette-based approach to detect RNA mutations and modifications. A phage RNA was used as a nanolatch that shifted between “latched state” and “unlatched state” during base pairing with different RNAs. As a result, different current fluctuations reflected the mutant RNA fragments involved in the reaction, offering new perspectives on epigenetic RNA sequencing.^96^ Additionally, sequencing of miRNA, long-coding RNA (lncRNA), circular RNA (circRNA), and tRNA has also been attempted.^97^^,^^98^^,^^99^^,^^100^ MinION has also been instrumental in the development of direct RNA sequencing.^101^^,^^102^^,^^103^^,^^104^

## Transition from DNA and RNA to protein

In April 2022, *Science* published a special issue to announce an unprecedented accomplishment by telomere-to-telomere (T2T) that the first complete, gapless sequence of a human genome sequence deciphered. This milestone filled the last remaining 8% gap of human genetic information left by the Human Genome Project.^105^^,^^106^^,^^107^^,^^108^^,^^109^ The long-read sequencing technologies, such as ONT, played an irreplaceable role in closing the previously unsequenceable gaps consisting of highly repetitive and complex regions of the human genome.^106^ Such accomplishment means that humans can now stroll in the DNA world. However, it is found that there are only 20,000 protein-coding genes in the human genome, much less than anticipated. This fact suggests that the complexity of human vital activity results from far beyond the genetic diversity.^110^ Consequently, guided by the central dogma, nanopore technology has strategically expanded its focus from DNA sequencing to direct RNA sequencing, and is now making significant inroads into the more challenging realm of protein sequencing, which logically follows the direct detection of PTMs.^111^ However, there are other factors that contribute to the protein complexity in disease evolution, such as epigenetics, alternative splicing of RNA transcripts, and PTMs.^112^ Taking Alzheimer’s disease (AD) for example, patients with AD rarely carry pathogenic genes, and the most typical pathological feature is the neuritic plaques in the brain tissue formed by extracellular aggregation containing amyloid beta peptides (Aβ).^113^ A mainstream interpretation for the pathogenesis is that AD is associated with excessive PTMs of Aβ, such as phosphorylation and glycosylation, which enhance the aggregation propensity or alter the interaction with neuronal cells of the Aβ_1-42_/Aβ_1-40_ peptides.^114^ Evidently, protein sequencing will become the next frontier in the field for exploring the pathogenesis and progress of human neurodegenerative diseases at the molecular level.^115^ At present, traditional protein sequencing methods are facing severe limits,^116^ and consequently, researchers are now enthusiastic to duplicate the success of nanopore DNA sequencing in the proteome.^117^^,^^118^

### Traditional protein analysis techniques

Protein sequencing essentially refers to decoding the primary structure, i.e., determining the amino acid sequence of all or part of a protein or peptide. Many severe chronic diseases, such as dementia, are caused by protein malfunctions. Thus, protein sequencing is crucial for early diagnosis and treatment, making protein *de novo* sequencing an urgent necessity. As nanopore technologies had fulfilled DNA sequencing, people were wondering whether nanopore could be a powerful tool for protein sequencing as well.^111^^,^^118^ However, it is straightforward to picture how challenging protein sequencing is, even if nucleic acids and peptides are both linear polymers.

On the one hand, proteins have complex higher-order structures that must be unfolded to expose their primary structures. Unlike nucleotides, there is a significant heterogeneity among amino acids, for example, charge and hydrophobicity, so driving protein molecules through a nanopore could be troublesome. On the other hand, amino acids are approximately one-tenth of nucleotides in size, which requires shrinking the nanopore aperture and slowing the translocation rate accordingly. Meanwhile, there are 20 proteinogenic amino acids, while there are only 4 nucleotides in DNA or RNA strands. To predict variations in amino acids along a peptide would be daunting, and considerable efforts are needed to develop proper algorithms.

Traditional protein sequencing methods include Edman degradation sequencing, fluorescent protein fingerprinting, Cellular Indexing of Transcriptomes and Epitopes by sequencing (CITE-seq), and Mass Spectrometry (MS). The Edman degradation sequencing method was first proposed in 1949. It is renowned for its high accuracy but hampered by low efficiency. Additionally, it is incompetent when dealing with mixed samples or proteins without any free α-amino group at the N-terminus, which is nevertheless common in detecting PTMs.^119^ In 2015, Swaminathan et al. pioneered the combination of Edman degradation with fluorescence technology to achieve specific recognition of single-molecule peptides in a mixture. The advantage of this approach lies in its high throughput, allowing parallel detection of millions of peptide molecules. However, it is time-consuming and costly because the removal of the N-terminal amino acid must be carried out by the Edman degradation as a pre-condition. Meanwhile, the fluorescent labeling efficiency is highly susceptible to the chemical reagents used in the Edman degradation process.^120^ In 2017, Stoeckius et al. proposed CITE-seq to identify cell surface proteins for the first time.^121^ CITE-seq is capable of efficiently collecting transcriptomic and proteomic data simultaneously, but it is also costly because various antibodies are rapidly consumed during sequencing. Besides, the acquisition of protein information depends on the binding robustness between antigens and antibodies, and the scope of application is limited to cell surface proteins only. In 2018, Chirlmin Joo et al. developed a remarkable fluorescent protein fingerprinting technology for protein sequencing at the single-molecule level.^122^ Unfortunately, not all the proteinogenic amino acids are able to be fluorescently labeled, and the sequencing accuracy greatly depends on the labeling efficiency.

MS is a highly established tool for protein analysis,^123^ and it can be generally divided into two categories: bottom-up MS and top-down MS.^124^ While bottom-up MS offers three key advantages: high accuracy, high throughput, and good repeatability, it suffers from short read length, high cost, narrow detection dynamic range, low sensitivity and 70% of spectra remaining unidentified.^124^^,^^125^^,^^126^ On the other hand, top-down MS is good at detecting the complete sequence of a protein, protein variants, and PTMs, in spite of drawbacks such as low throughput, detection limited to molecules less than 70 kDa, and a reduction in sensitivity for protein than polypeptide.^127^

Strictly speaking, MS is closer to protein classification rather than sequencing at this time.^127^^,^^128^ Meanwhile, nanopore technology has emerged as a cutting-edge tool for DNA sequencing due to low cost, label-free, high throughput, long read length, single-molecule detection, and real-time sequencing. Besides DNA sequencing, researchers have been attempting to utilize nanopores for peptides and proteins detection. Nowadays, nanopore technology has demonstrated potential for proteomics, including protein *de novo* sequencing, PTM/mutation detection, peptide biomarker identification, protein-protein interaction (PPI) measurement, and nanopore enzymology, and so forth. The following will elaborate on the latest progress on nanopore single-molecule technology in proteomics.

## Nanopore protein sequencing

**Figure 2:**
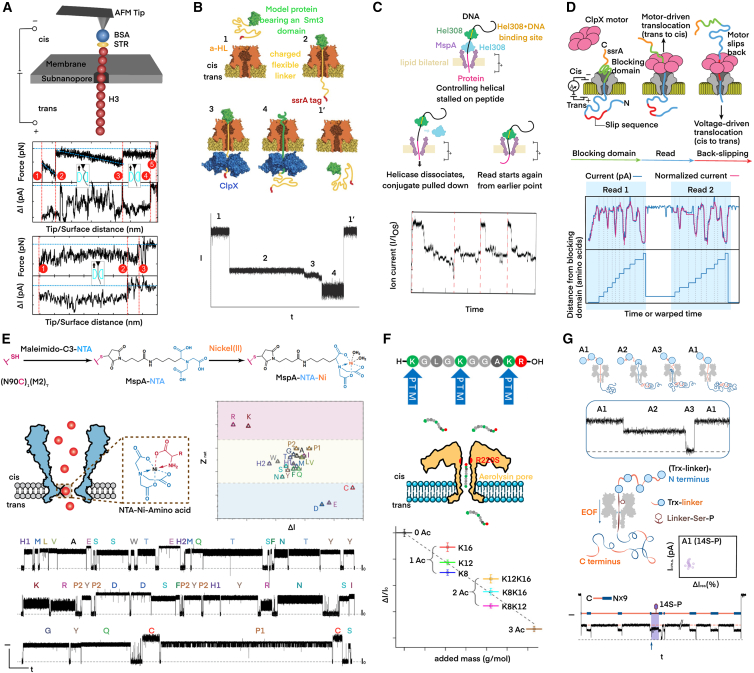
Nanopore protein sequencing and PTM detection (A) Peptides chain was tethered to the cantilever of an AFM to control the translocation speed. The periodical fluctuations indicated the residual information. Reproduced with permission from ref.^131^ (B) Biological nanopores implements analogous approach utilized in DNA sequencing to sense amino acids with ClpX binding to the ssrA sequence and dragging the protein tail from *cis* to *trans*. Reproduced with permission from ref.^132^ (C) Researchers conducted peptide-oligonucleotide conjugates for single amino acid identification and demonstrated “rewind” peptide reads (from *trans* to *cis*) to reduce error rate. Reproduced with permission from ref.^133^ (D) More streamlined rewinding process was realized by adding the ClpX motor to the *cis* solution to pull the peptide back after it was threaded into the nanopore by electrophoretic force. Reproduced with permission from ref.^134^ (E) The unambiguous discrimination of all 20 proteinogenic amino acids reported by Nature. The Ni-NTA modified MspA nanopore finally achieved 98.8% accuracy and could precisely predict along intact peptide chains without requiring enzymatic or chemical cleavage. Reproduced with permission from ref.^135^ (F and G) Recognition of PTMs by nanopore technology. (F) showed that the mutant AeL nanopore could distinguish the variety of acetylated and methylated lysine residues in human histone H4 and its derived peptide. Reproduced with permission from ref.^136^ (G) showed the deep-site profiling of PTMs in long-chain polypeptides (>1,200 residues). Reproduced with permission from ref.^137^

So far, researchers have adopted different countermeasures to unfold proteins and facilitate the peptide translocation through nanopores, analogous to DNA. Among them, the solid-state nanopore has first made remarkable progress. Timp et al. took advantage of the solid-state nanopores to resist protein denaturants, pioneering the use of surfactant agent (sodium dodecyl sulfate, SDS) and reducing agent (β-mercaptoethanol, BME) to denature proteins. SDS anions were uniformly attached to the peptide chain so that the electrophoretic mechanism would work as if it were dealing with a nucleic acid chain. To improve the precision, a tightly focused electron beam was used to sputter pores with a sub-nanometer diameter in an inorganic thin film. Through control experiments involving homopolymer, block co-polymer, native, and modified peptides, it was found that these subnanopores were capable of probing the conformational variation that led to volume changes of a minimum of 0.07 nm^3^, even though the pore constriction accommodated 4-6 adjacent residues at the same time. This nanopore strategy not only reads a protein’s primary structure but also proposes an approach for detecting single-site PTMs.^129^ This work was considered a proof-of-concept for nanopore protein sequencing, although parsing the full diversity of the amino acid alphabet remained uncertain.^130^ Furthermore, to systematically control the translocation kinetics of peptide chains in a subnanopore, Dong et al. tethered the denatured proteins to the cantilever of an AFM and threaded single molecules through the subnanopore at a constant speed (Figure 2A), 4 nm/s, which is nearly 10,000 times slower than free translocation, and thereby the read fidelity was adequate for discriminating two histone protein variants at the single-molecule level. This is the first time of measuring the force and current concurrently during a peptide translocating through a nanopore, so that the role SDS played was experimentally confirmed.^131^ Later on, Dekker et al. disclosed how SDS assisted protein to transport and improved the solid-state nanopore’s read length,^138^ and the Timp team managed to measure the sizes of fully dehydrated metal cations by utilizing the noise characteristics in the ionic current from subnanopores.^139^ An up-to-date study stated that current blockades from the translocation of two proteins through subnanopores were classified at the single-molecule level by deep learning. The consensus from clustering hundreds of label-free single-molecule signals resolved the primary structures of Aβ_1-42_ and its variants with single-residue spatial resolution after the membrane thickness was reduced to 5 nm and the sampling rate was increased to 500 kHz. Consequently, important PTMs and mutations, such as phosphoserine and the Arctic mutation E22G, were detected with site-specificity.^140^

Protein unfolding strategies for biological pores include temperature regulation,^141^ pH value adjustment,^142^ and attachment of oligonucleotides to the ends of proteins.^143^ Recently, enzymes have been recognized as regulatory tools for protein unfolding and translocation in biological nanopores. For example, ClpX, an AAA+ protein, can bind to the ssrA tag, unfold proteins in the Smt3 domain, and facilitate the unidirectional, stepwise translocation of peptide chains through engineered nanopores (Figure 2B).^132^ However, irregular stepping behaviors of ClpX disrupt the ratcheting motion of peptide chains, making it difficult to obtain proper nanopore readouts for sequencing. To overcome this flaw, researchers have explored alternatives. Phi29 DNA polymerase and Hel308 DNA helicase were practicable after constructing peptide-oligonucleotide conjugates or expanding enzyme loading sites. These schemes alleviated random errors in long reads and enabled the identification of single amino acid mutations (Figure 2C).^133^^,^^144^ In 2024, *Nature* published a novel method by leveraging the ClpX unfoldase to reread long protein strands through a CsgG nanopore.^134^ As shown in Figure 2D, the protein complex comprised an unstructured, negatively charged N-terminal sequence that was attached to a stably folded domain (Smt3) while the ssrA tag was fused to the C-terminus. Once the analyte was captured by electrophoresis for an incomplete translocation to the *trans*-side, the ssrA tag begun to bind to ClpX in the *cis*-side. Finally, the unfoldase retracted the sequence by unidirectionally ratcheting for the nanopore to reread the same protein strand multiple times, thereby laying the foundation for high-throughput nanopore protein sequencing due to its compatibility with the MinION platform. This advancement is a complement to proteomics, facilitating the detection of single amino acid substitutions and PTMs across protein strands up to hundreds of residues in length at the single-molecule level. It is worth mentioning that Wanunu et al. described an enzyme-free method to achieve unidirectional, slow transport of full-length proteins through nanopores with concentrated GdmCl buffer. GdmCl not only facilitated protein unfolding but also generated stretching/driving force for an unstructured peptide chain in an α-HL pore through electroosmotic flow, resembling the translocation dynamics of ssDNA.^145^

Besides the endeavor to thread proteins through nanopores, considerable efforts have been made to identify all 20 proteinogenic amino acids using nanopores. In 2017, Asandei et al. proposed an interesting design of synthetic peptides, which introduced two oppositely charged amino acid fragments to each side of the analyte sequence. This significantly extended the residence time of the peptide in the pore so that the difference between alanine and tryptophan in volume was perceived by an α-HL nanopore.^146^ Similarly, Ge et al. synthesized bipolar peptide probes by linking four aspartic acids and five arginines to the N-terminus and C-terminus of individual amino acids, respectively. The probe interacted with the inner wall of an aerolysin nanopore and generated characteristic current blockades that could be used to recognize nine proteinogenic amino acids.^147^ Oukhaled et al. took a step forward with a short polycationic carrier and identified 13 proteinogenic amino acids using aerolysin nanopores.^148^ Huang et al.’s work was considered a milestone.^135^ An MspA nanopore containing a sole Ni^2+^ modification at the constriction was capable of simultaneously discriminating twenty proteinogenic amino acids and their four PTMs. It was the first time that all 20 proteinogenic amino acids were unambiguously discriminated (Figure 2E). The accuracy reached 98.6%, assisted by machine learning, and no carrier was involved. Immediately after that, Zhang et al. constructed a Cu^2+^-functionalized MspA nanopore.^149^ With the N91H substitution, such engineered nanopores also achieved direct identification of all 20 proteinogenic amino acids when a machine learning algorithm was applied. Besides informing PTM and unnatural amino acids, this strategy was able to distinguish peptides with single amino acid replacements through hydrolysis and provided clues to infer the sequence of targeted peptides based on the capture rate of cleaved amino acids in real-time. Almost the same time, Zhang et al. reported that wild-type together with (M113F)wi_7_ α-HL nanopores could also identify every proteinogenic amino acid in a specified position of a phenylalanine-containing peptide and sequence a peptide based on the relative abundance of amino acids in the mixture through enzymatic cut when a homemade peptide probe was used.^150^ Above techniques are summarized in Table 1.

**Table 1:** Overview of advances in nanopore-based protein sequencing

Methods	Principles	Pros	Cons	Prospects	Reference
Solid-State nanopore	E-beam sputtered subnanopore in ultrathin film; SDS-assisted translocation.	Capable of reading protein primary structure; Site-specific detection of PTM and mutation.	Incapable at the single-molecule level; Not applicable to unknown sequence.	Proof-of-concept for nanopore protein sequencing; MD simulations and AI algorithm upgrade.	Kennedy et al.^129^ Restrepo et al.^138^ Chen et al.^140^
Solid-state nanopore with AFM manipulation	Denatured protein tethered to the AFM tip was captured and pulled out of the subnanopore slowly.	Concurrent measurement of force and current; Discriminate protein variants by a single read.	Low yield rate and read fidelity; High complexity and cost.	Potential for *de novo* sequencing and re-reading the same molecule.	Dong et al.^131^
Ratcheting peptide-oligonucleotide conjugates	Phi29 DNA polymerase controls the translocation of the DNA handle attached by a peptide.	Detect single residue substitution; Conjugates both N- and C-termini of peptides	Limited read length (∼14 nucleotides); Low read fidelity (accommodating 5–6 amino acids).	The first ratcheting in protein sequencing that propels enzyme-related studies	Yan et al.^144^
Multiple re-reads of a single protein	DNA-peptide conjugate is pulled through the MspA nanopore by the DNA helicase Hel308	Pulling in observable steps (half-nucleotide ∼0.33 nm); Low error rate (<10^−6^) after 30X coverage.	Low fidelity in single read and require a number of re-reads; Intrinsic read length (∼25 amino acids).	Promising for developing a low-cost, high-throughput commercial system.	Brinkerhoff et al.^133^
Enzyme-mediated protein unfolding	ClpX motor prebound to protein facilitates protein unfolding and stepwise translocation.	Long read length (hundreds of residues); Multiple re-reads; Full-length reading of the model protein.	Low accuracy; Low throughput; Difficult for native protein sequences.	Potential for using the unfoldase motor is inactivated until the protein strand is captured.	Nivala et al.^132^ Motone et al.^134^
Enzyme-free protein unfolding	GdmCl buffer unfolds proteins and enhances EOF to drive translocation.	Unidirectional protein translocation; Engineered nanopores are not necessary.	Lack precise control; Require harsh chemistry.	Enzyme-mediated unfolding may prevent strand jamming.	Yu et al.^145^
Peptide probes-assisted amino acids identification	Synthetic peptides carry amino acids to translocate and enhance the signal.	Long dwell time events (20–200 ms); Identify individual amino acids; Peptide sequence.	Limited to engineered single amino acids; Only for short purified peptides.	Promising for parallel protein sequencing if cooperating with enzymes such as ClpX.	Asandei et al.^146^ Ge et al.^147^ Ouldali et al.^148^ Zhang et al.^150^
Engineered nanopores for discriminating amino acids	Nanopores modified by metal ions interact with amino acids and create distinct current blockades.	High accuracy; Capable of detecting PTMs; Consecutive identification of cleaved amino acids.	Limited to short purified peptides; Poor at solving folding structural heterogeneity.	Considered as a milestone and pave a way for nanopore protein sequencing.	Wang et al.^135^ Zhang et al.^149^

## Nanopore for proteomics beyond sequencing

### Post-translational modification detection

PTMs refer to the covalent modifications of one or multiple amino acids during or after protein translation. There are more than 300 known PTMs, which commonly include phosphorylation, glycosylation, ubiquitination, nitrosylation, methylation, acetylation, lipidation, and so forth. PTMs are so prevalent in proteins that they may enhance the function, expand the diversity, increase the complexity, and regulate the activity.^151^ How to go about precisely mapping PTMs is of great interest. Since high-resolution MS is the classical method widely adopted to analyze fragmented proteins, but it has limitations in detecting PTMs in a complex environment with low-abundance samples. In contrast, nanopore has unveiled itss potential for PTMs detection at the single-molecule level.

In 2014, Larrea and Bayley succeeded in distinguishing unphosphorylated, monophosphorylated, and biphosphorylated peptides.^152^ They selected a set of mutant Trx proteins with several adjacent PKA phosphorylation sites near the C-terminus. By attaching a short DNA sequence to the C-terminal cysteine, the C-terminal domain was unfolded. Then, the remaining part was forced to unfold after being captured by a nanopore. Peptides with different phosphorylation states were distinguished according to their specific ionic current levels and the electric noise during each current blockade as the C-terminal sequence translocated through the nanopore. Rosen et al. showed that nanopore-based phosphorylation detection usually required either primer-driven substrate or additional nanopore modifications to amplify the current signal.^153^ To overcome this limitation, Li et al. chose a charge-sensitive AeL nanopore.^154^ By mutating residues such as T232K and K238Q in the pore, they detected an unmodified phosphorylated tau peptide. T232K mutation formed an electrostatic trap, thereby enhancing electrostatic interaction, while the K238Q mutation increased the repulsion barrier for the Tau fragment. This combination effectively extended the residence time of the peptides in the pore, enabling Tau phosphorylation identification. Since hyperphosphorylated Tau is abundant in AD patients' cerebrospinal fluid and current assays are costly, non-specific, and unstable, the nanopore-based assay in this study shows a promising clinical diagnostic alternative. Building on these advances, Dekker et al. used Hel308 helicase docked to the rim of MspA nanopore to pull the peptide-oligonucleotide conjugate.^155^ The peptide would slowly translocate in ∼0.3 nm steps by the helicase, and individual amino acid substitutions were scanned with high accuracy. With this method, phosphorylated sequences with one or two closely spaced residues could be clearly discriminated. Subsequently, the team optimized the technique and analysis algorithm so that not only phosphorylation was detected, but also sulfation modifications on peptide hormones were identified.^156^ Moreover, sulfation and phosphorylation could be distinguished on the same residue with accuracy over 90%. In terms of specifying adjacent modification sites, the positioning accuracy achieved 96%.

Previous studies have also shown that nanopore is capable of detecting glycosylation in proteins.^157^ Some scholars have pointed out that studies at early stage only used synthetic peptides, bypassing certain typical challenges associated with natural peptides, such as rapid translocation and proteolytic cleavage. For example, the glycosyl groups on natural glycopeptides are hydrophilic, which causes peptides to translocate too fast to be analyzed by nanopores. It is also worth noting that K^+^ can gather near FraC D10 to form an electrostatic barrier.^158^ Fahie and Chen modified the flexible loop on outer membrane protein G (OmpG) by attaching a biotin ligand to it.^159^ The experimental results showed that when biotin ligands bound to the affinity on the glycopeptide, leading to changes in the gating parameters induced by the flexible loop 6 surface charge differences. Thus, the glycosylated and deglycosylated affine isomers were distinguished.

Wloka et al. demonstrated that ClyA nanopores were capable of discriminating ubiquitinated and polyubiquitinated proteins.^160^ ClyA nanopores not only distinguished isomeric monoubiquitin proteins, but also provided real-time observation of protein ubiquitination of 20–50 kDa under physiological conditions. It is claimed that nanopores with wider diameters were more suitable for monitoring the ubiquitination process. After optimizing the pore size, biological nanopores managed to monitor and analyze protein ubiquitination *in vitro*. In addition, Nir et al. proved that solid-state nanopores could also distinguish ubiquitin monomers and short ubiquitin polymers at the single-molecule level by adjusting the buffer pH to slow down the translocation speed of the analyte.^161^

Nanopore can also detect some rare types of PTMs or simultaneously monitor two or more PTMs that are difficult for MS.^162^ Hu et al. used 11-nm-diameter SiN_X_ nanopores to investigate the effect of two ubiquitinated histones (ubH2A and ubH2B) on nucleosome stability.^163^ It was found that ubH2B had a weaker effect than ubH2A, and the potential of solid-state nanopores in probing PTMs on ribosome dynamic structure was demonstrated. Recent studies have revealed that PTMs can be recognized as specific labels for peptides with identical mass. Ensslen et al. adopted the R220S mutant AeL nanopore as shown in Figure 2F, and successfully distinguished peptides derived from human histone H4 protein based on the variety of acetylated and methylated lysine residues,^136^ which highlights the potential of nanopore for identifying protein isoforms that cannot be handled by MS.

By modeling the interaction between hydroxylated proline and its specific recognition protein, Chang et al. introduced a hydrophobic region with the Y224/T274W mutation into the lumen of aerolysin nanopore,^164^ which enabled highly sensitive recognition of proline hydroxylation on the HIF-1α fragment even in the presence of adjacent oxidation. Some studies have shown the competitive interactions between phosphorylation and glycosylation at serine/threonine residues,^165^^,^^166^ and there is a trade-off phenomenon in patients with AD. The decreasing glycosylation level and hyperphosphorylation state are closely related to the accumulation of the pathogenic tau proteins. To solve this problem, Pérez et al. designed a bipolar model peptide to distinguish phosphorylation from glycosylation without labels,^167^ FraC nanopore was modified with negative charge at the N-terminus and positive charge at the C-terminus, along with a serine at position 11 prone to be phosphorylated or glycosylated. Notably, Martin-Baniandres et al. presented a novel enzyme-less method based on a mutated α-HL nanopore (NN-113R)_7_.^137^ In stead of enzymatic reaction, Figure 2G demonstrated that the Trx-linker concatemers would be caught by a charge-selective nanopore and achieved EOF-driven concatemer translocation through the nanopore. The characteristic repeating current recordings indicated the PTM information within a polyprotein of over 1,200 residues. Additionally, Lan et al. employed a phosphorylation-specific chemical binder called Phos-tag dizic, which selectively binds to monophosphates and amplifies the phosphorylation signal, and managed to differentiate phosphorylation from similar PTMs such as glutathionylation.^168^

### Biomarker detection

Biomarkers are currently a major clinical focus as they serve as crucial indicators for clinical diagnosis, and more importantly peptide biomarkers are found closely related to the pathogenesis of certain chronic diseases.^169^ Changes in the concentration of analytes in body fluids can reflect the physiological or pathological state of the organism, which makes the composition of peptides an important characteristic for disease diagnosis. In addition, the progress of peptidomics holds great significance for discovering potential biomarkers and therapeutic regimen.^170^ Nanopore is regarded as one of the most suitable approach for detecting peptide markers in body fluids, such as urine, plasma, cerebrospinal fluid and saliva, due to its advantages of being label-free, high throughput, high specificity, low cost, and adaption for low-abundant samples under complex circumstances.^171^ Generally, there are two common methods to measure peptide markers in mixture through nanopores: (1) directly sense peptides and (2) indirectly sense peptides, for example, using specific aptamers, forming chemical bonds, or through antigen - antibody interactions.

**Figure 3:**
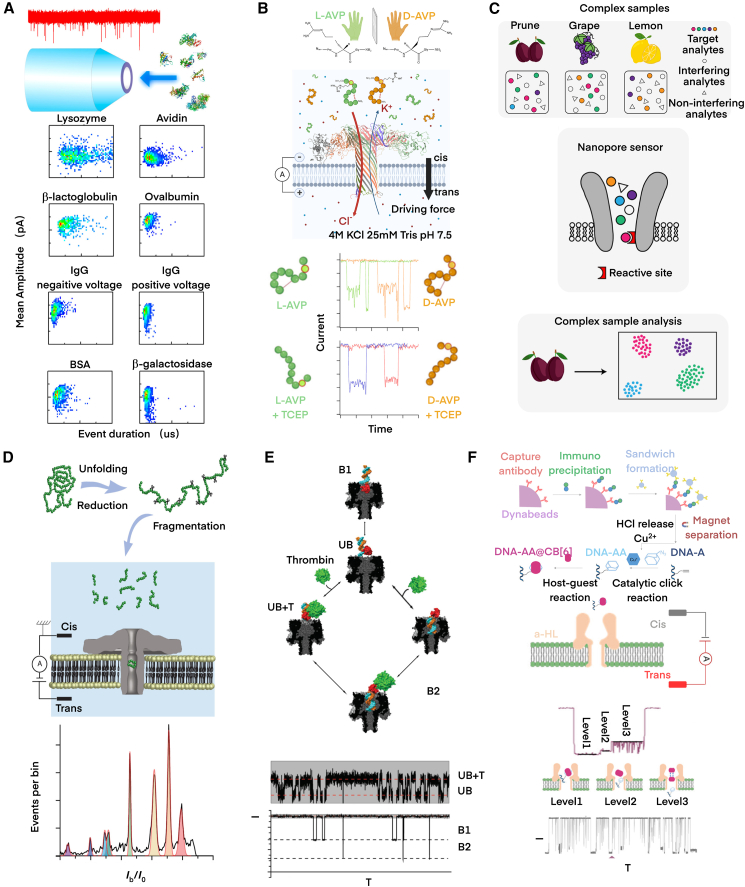
Nanopore biomarker detection (A) Quartz nanopores facilitated label-free discrimination of diverse proteins, representing a low-cost, high-throughput nanopore sensing approach. Reproduced with permission from ref.^174^ (B) Wild-type aerolysin nanopore was utilized to directly detect protein/peptide enantiomers which was able to distinguish L-AVP and D-AVP. Reproduced with permission from ref.^175^ (C) With aptamer-modified pore lumen, MspA-PBA nanopore identified *cis*-diol isomers (citrate/isocitrate, fructose/glucose) through distinct current signatures. Reproduced with permission from ref.^176^ (D) Cleaved native proteins generated unique current information during nanopore translocation, with capture frequency correlating with protein concentration. Reproduced with permission from ref.^177^ (E) Attachment of probes to the nanopore exterior facilitated immobilizing and sensitive thrombin quantification. Reproduced with permission from ref.^178^ (F) Indirect peptide detection by participating in chemical reactions and releasing intermediates prior to nanopore sensing. This method is always utilized when biomarkers have ultralow abundance and need signal amplification. Reproduced with permission from ref.^179^

Many studies have shown that nanopores can directly detect peptide markers.^172^^,^^173^ Li et al. demonstrated that glass nanopores could detect proteins of varying molecular weights from lysozyme (14 kDa) to β-galactosidase (465 kDa) as illustrated in Figure 3A, which included the first detection of mammalian prion protein.^174^ Chen et al. further demonstrated that reducing the glass nanopore diameter and increasing wall thickness would enhance the SNR, and the current changes during DNA translocation depended on the salt concentrations and pH.^180^ Furthermore, previous studies have shown that the nanopore electro-osmotic trap (NEOtrap) can capture and identify single proteins based on their mass, size, and shape.^181^^,^^182^^,^^183^^,^^184^ However, NEOtrap has problems with tiny proteins due to their extremely short capture time.^185^ Wen et al. recently upgraded the system and proposed NEOtrap 2.0 with enhanced capture capabilities.^186^ A cholesterol molecule was connected to one end of the double helix of the DNA-origami sphere and used as an anchor to bind the DNA-origami sphere to the nanopore. Cholesterol-induced binding not only inhibited the fluctuation of spheres and the escape of proteins from the pores, but also improved the capture time and SNR. In addition, it was found that if the DNA-origami sphere was vertically aligned, the EOF became stronger and therefore enhanced the sensitivity. Eventually, the NEOtrap 2.0 was extended to proteins as small as 14 kDa.

Wild-type biological nanopores are capable of detecting biomarkers when the pore dimensions and chemical conditions are appropriate. The intrinsic electroosmotic flow within OmpF nanopore generates a robust driving force. Based on this feature, Gao et al. constructed a direct single-molecule oligosaccharide sensing platform for high-precision identification of different oligosaccharide isomers assisted by a supervised machine learning algorithm.^187^ Li et al. used the aerolysin nanopore to detect fifteen steviol glycosides and pioneeringly proposed that two types of glycans differing by only one hydroxyl group become easily distinguishable at high voltage gradient.^188^ Ratinho et al. employed wild-type aerolysin nanopore to detect the enantiomers of antidiuretic hormone (L-AVP and D-AVP) and their conformational changes.^175^ Figure 3B demonstrated that L-AVP and D-AVP generated blocking current signals with signatures, allowing for their discrimination. Moreover, Grieve et al. utilized aerolysin nanopore to identify bradykinin (BK) and des-Arg9-bradykinin (DABK) in blood,^189^ and Versloot et al. used Cykt and ClyA nanopores to identify isomeric peptides, including structural isomers, enantiomers, and diastereomers, all of which cannot be distinguished by MS yet.^190^

Although nanopores have presented the capability of distinguishing almost identical biomarkers, there exist certain proteins or biomolecules that cannot translocate through the nanopore directly owing to their overly large dimensions or the presence of a negative charge. As a possible solution, Huang et al. proved that a large biological nanopore was helpful to identify and distinguish large folded proteins, and the competition between EOF and EPF would promote the protein to stay in the pore for a longer time.^191^ Lately, researchers tended to implement nanopore modification to fit with large negatively charged proteins, such as bovine serum albumin (BSA), human serum albumin (HSA), and human transferrin (HTR), which cannot overcome the entropy and electrostatic potential energy to enter the pore themselves. For instance, reducing the EOF by introducing a positive charge inside the pore constriction generated an electroosmotic vortex and facilitated the entry of large folded proteins into the pore. Moreover, the retention time of HSA was significantly longer than that of HTR, achieving the effect of distinguishing between two human plasma proteins.^181^ Sauciuc et al. achieved the translocation of nonuniformly charged polypeptides by engineering the CytK nanopore lumen with four negatively charged aspartic acid residues. The 2E-4D-CytK design represented a strategic advancement, combining ion selectivity with mechanical control to enable high-resolution protein analysis.^192^

Fan et al. demonstrated a phenylboric acid-modified MspA nanopore (MspA-PBA) was capable of discriminating up to 12 kinds of *cis*-diols simultaneously.^176^ Figure 3C is a schematic for the MspA-PBA nanopore to differentiate isomers, including citric acid and isocitric acid, fructose and glucose, and the enantiomer DL-malic acid. Their results have reached an accuracy of 99.3% with the aid of machine learning, which suggested potential of direct application for portable food industry testing. Subsequently, Lu et al. utilized the T240R-mutated aerolysin nanopore and introduced the R-binaphthyl tag into glycans to conduct the structural analysis of neutral glycans, which resulted in the discrimination of six disaccharide isomers.^193^ Zhang et al. found a nanobody Ty1 that could form a multivalent interaction with trimeric SARS-CoV-2 Spike protein, and attached Ty1 to ClyA nanopore with an ssDNA linker. The pores modified with Ty1 were capable of stochastic sensing of Spike protein, insusceptible to the complex environment in the serum.^194^

In addition, a nanopore can facilitate indirect detection of proteins by analyzing fragments after enzyme-catalyzed cleavage.^195^ In clinical applications, a rapid and sensitive detection method of toxin-like peptides is urgently needed. For example, Wang et al. showed that botulinum neurotoxin type B (BoNT-B) could present an enzymatic mechanism when digesting the substrate synapsin SB2. The enzymatic reaction could be indirectly monitored by counting the catalytic product through AeL nanopores, i.e., the toxin BoNT-B in serum at the sub-nM scale within minutes.^196^ Recently, Bakshloo et al. showed that polypeptide fragments from myoglobin, lysozyme, and cytochrome *c* cleaved by trypsin could translocate AeL nanopores and generate distinct I_b_/I_0_ signals so that different natural proteins were fingerprinted accordingly.^177^ This method could distinguish various proteins regardless of their size, conformation, and charge, and the capture efficiency of peptide fragments was proportional to their concentrations (Figure 3D).

Indirect sensing can also be achieved by attaching molecular aptamers (e.g., short DNA strands or peptides) to nanopores or analytes. In 2012, two teams connected DNA sequences to α-HL nanopore and ClyA nanopore, respectively, and realized the detection of thrombin.^178^^,^^197^ Consider Figure 3E as an example, a 15-mer DNA oligoA was attached to the entrance of the N17C mutant α-HL pore via a disulfide bond. When the aptamer interacted with thrombin, the variation in current levels was intuitive. Moreover, ESAT-6/CFP-10 is a pair of proteins from *Mycobacterium tuberculosis* and is naturally low-abundant in serum. Wang et al. converted ESAT-6/CFP-10 signal into released Cu^+^ using nanopore-based click chemistry.^179^ When the DNA-azido adamantane (DNA-AA) complex was formed by Cu^+^ and passed through an α-HL nanopore, a featured signal was recorded, and thereby ESAT-6/CFP-10 was indirectly quantified (Figure 3F). Similarly, Bell et al. and Sze et al. successively connected DNA vectors to glass nanopores so as to identify proteins in a mixed solution.^198^^,^^199^ Meanwhile, proteins can also work as aptamers.^200^^,^^201^ Foster et al. performed flow cytometry-based high-throughput screening of OmpG nanopores containing peptide motifs and found that loop 3 or 6 had strong fluorescent signals suitable for further single-channel analysis. They connected FLAG to loop 3 and SA1.1 to loop 6 to construct an OmpG nanopore with a dipeptide motif, and specifically detected FG4R and streptomycin in the mixed solution.^202^ Besides, Galenkamp et al. reported a ClyA nanopore with internal protein aptamers SBD1 and GBP, which detected asparagine and glucose in body fluids and monitored the concentration of small metabolites dynamically.^203^ This method simplified the sample preparation process and reduced the required volume of body fluids. Furthermore, chemical groups can be involved.^204^^,^^205^ For example, attaching the nitrilotriacetic acid group inside a metalized silicon nitride nanopore could form a stable bond with His6-tagged protein molecules, and a solid-state nanopore with His6-tagged protein inside specifically recognized IgG class protein.^206^ At last, Wang et al. demonstrated that antigen molecules could be used as probes to identify antibodies in mixed solutions and homologous antibodies.^207^

### Protein-protein interaction detection

PPIs underpin fundamental cellular function under both normal and pathogenic conditions, and are crucial for drug design, such as small molecule inhibitors. Unfortunately, PPI is a transient process, the observation of which is challenging. Recently, nanopore technology has demonstrated the potential to monitor the interaction between proteins and nucleotide ligands,^208^ and quantify the reaction tendency by the association constant K_on_ and dissociation constant K_off_ between protein and nucleotide ligands.^178^ Strong evidence has shown that nanopores can further characterize PPIs even dynamically.^209^^,^^210^^,^^211^^,^^212^

**Figure 4:**
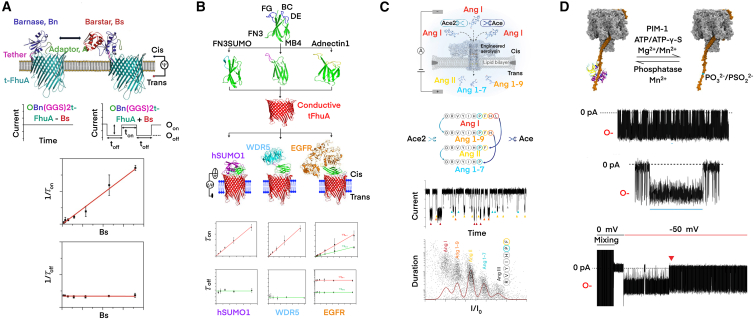
Nanopore detection of PPIs and enzymology (A) Nanopore was engineered for real-time sampling of transient PPIs. The current gating events demonstrated the reversible capture of the target protein. Reproduced with permission from ref.^214^ (B) By engineering three distinct domains, each specifically binding to different target proteins, the simultaneous detection of three PPIs was achieved with one aptamer. Reproduced with permission from ref.^215^ (C) The T232K aerolysin nanopore exhibited accurate discrimination of Ang peptides differing by a single amino acid, thus revealing the crosstalk effect between enzymes ACE1 and ACE2. Reproduced with permission from ref.^216^ (D) The α-hemolysin nanopore was used with a phosphorylated aptamer to monitor the phosphorylation and dephosphorylation process on the single-molecule substrate. Reproduced with permission from ref.^217^

Previously, PPIs were detected by connecting a protein receptor to a solid-state pore to bind the target protein.^206^^,^^213^ However, the anchor site of the receptor was out of control, which compromised the performance. It is believed that there are two pre-conditions before applying nanopores to PPI studies. Firstly, PPIs must occur outside the lumen of the nanopore to prevent permanent channel blockage by protein-protein complex. Secondly, the reversible association and disassociation activities must be translated into nanopore electrical signals. Therefore, Thakur et al. constructed Bn (GGS)_2_t-FhuA (truncated FhuA) and t-FhuA (GGS)_2_Bn nanopores and detected the transiently reversible PPI between Bn and its specific inhibitor Bs in mammalian serum.^214^ Through the linker (GGS)_2_, the aptamer was close to the aperture of the nanopore to improve the binding affinity with the target protein, and the affinity was enhanced as the Bs concentration increased while the Bn site was not saturated (Figure 4A). Thereafter, Ahmad et al. tethered the same (GGS)_2_ linker for different protein recognition elements to the N-terminus of tFhuA nanopore.^215^ Three nanopore sensors for hSUMO1, WDR5, and the extracellular domain of EGFR were built, and characteristic electrical signals that represented each protein were observed (Figure 4B). Additionally, Oh et al. analyzed the PPIs between the p53TAD1 peptide and MDM2 using AeL nanopores in the tens of pM range.^218^ Oh et al. further demonstrated that biological nanopores could be developed into an ultrasensitive platform for rapidly screening small fractions of PPI inhibitors.^219^ Recently, Mayse et al. precisely monitored the binding and dissociation events between Myc and WDR5.^220^ A 13-residue-long MycWBM peptide ligand (QEDEEEIDVVSVE) was attached to the N-terminus of tFhuA nanopore via a flexible spacer, and the quantitative analysis of WDR5 was achieved.

Nowadays, PPIs are generally observed through the interaction between the analyte and the pore. Li et al. developed the funnel-shaped ClyA nanopores as molecular tweezers, which captured Abelson (Abl) kinase for a sufficient time to observe its conformational dynamics and the multiple states of Abl kinase interacting with ligands.^212^ To dynamically detect the origin of π-π interactions, Hao et al. constructed the (M113A)_7_ α-HL nanopore by the site-directed mutagenesis of the amino acid residues at specific positions within the pore.^221^ Such a mutant facilitated the smooth entry of Cucurbit [7] uril into the nanopore and formed a stable nanocage structure by non-covalently binding to the inner surface.^221^

### Enzymology analysis

Enzymes, which can be proteins or RNAs, are indispensable for biological catalytic reactions. In the field of biocatalysis, screening enzyme activity to monitor the kinetics of enzymatic reactions or identify the enzyme’s preferred substrate has long been a subject of great interest.^222^ Nanopore enzymology is an emerging field that utilizes nanopore sensors to study enzymatic processes at the single-molecule level.^223^ A fundamental requirement for nanopore enzymology is to capture and detect enzymes within an enzyme-favorable system so that we can characterize the kinetics of enzymatic reactions in forms of nanopore signals. Usually, such detection is indirect. Peptides are cleaved by specific enzymes before translocation,^224^ and the corresponding enzymatic reactions are disclosed by the current changes produced by translocations of pepsin,^225^ HIV-1 protease,^226^ trypsin,^227^ renin protease,^228^ and ClpX protease,^229^ and so on.

Recently, Jiang et al. applied genetic engineering to modify nanopores.^216^ The mutant T232K-AeL nanopore unambiguously revealed the crosstalk between the enzymes ACE1 and ACE2 in the renin-angiotensin system (Figure 4C). Yang et al. replaced the residue D56 of MspA with the unnatural amino acid AzK and constructed M2 MspA-D56AzK nanopores with azide functionalization.^230^ They elaborated that lysozyme attached to the pore rim could be continuously trapped in the vestibule and produce three different levels in current blockades. Similarly, Martin et al. incorporated two hydrolytic active sites into the FraC protein to match those of PETases.^231^ The assembled FraCm1/m2 nanopores were capable of deconstructing sub-micro- and nano-sized polyethylene terephthalate (nPET), revealing the potential for fast filtering, capturing, and decomposing.

Increasing evidences have suggested that nanopore technology possesses unique advantages over traditional kinase/phosphatase assays.^232^^,^^233^^,^^234^ Figure 4D illustrated that an engineered α-HL nanopore could be an excellent sensor for detecting label-free, single-molecule phosphorylation and dephosphorylation.^217^ Given that kinases and phosphatases induce phosphorylation and dephosphorylation phenomena of oligonucleotides/peptides, Ying et al. monitored the poly(DA)_4_ oligonucleotide kinase (PNK) phosphatase activity, and the process of PNK-catalyzed dephosphorylation of oligonucleotides was observed.^235^ In infectious disease research, Marx et al. developed single-molecule picometer-resolution nanopore tweezers (SPRNTs) to explore the detailed mechanism underlying the movement of SARS-CoV-2 helicase nsp13 along DNA and its inhibition mode.^236^

## Discussion

### Current limitations and challenges of protein sequencing

Although having offered unique potentials, nanopore protein sequencing is still immature. Significant improvements are needed in many aspects, such as native protein unfolding, translocation kinetics of peptide chains, and recognition of single amino acids along peptides. Thus, diverse strategies have emerged to address these issues, including enzymatic or chemical unfolding of proteins to realize protein full-length translocation through nanopores.^132^^,^^237^^,^^238^ Nevertheless, unfolding proteins in nanopore systems can be non-cooperative at a high denaturant concentration.^239^ Also, keeping protein molecules unfolded is challenging due to the influence of the electric field, temperature, and pH in the ambient environment.^154^^,^^240^ Given that the translocation of the peptide chain is mediated by both EOF and EPF, voltage bias and electrolyte pH can be used to regulate the molecule translocation speed, but with a lack of precision.^241^ Encouragingly, engineered nanopores can slow down the peptide translocation and acquire detailed conformational information.^242^^,^^243^ Additionally, the 2D MoS_2_ nanopore has presented the capability of accommodating only two amino acids per read.^244^ Besides driving the protein through the nanopore, identifying amino acids along peptide chains is another daunting challenge. Although numerous technical breakthroughs have been made, decoding a native protein sequence is still difficult. Ideally, a nanopore system that can function continuously with an unfoldase prebound to the analyte but prevented from initiating unfolding activity until the protein strand is captured by the nanopore is desired,^134^ which will perfectly duplicate the strategies developed for nanopore DNA sequencing. However, it is hard to implement due to inadequate experimental yield and incompetent readout accuracy. Furthermore, even if adjacent amino acids and site-specific PTMs or mutations in protein strands could be sequenced, the structural heterogeneity and complexity may still hold a significant challenge for nanopores to overcome. For example, resolving the precise architecture of N-glycans attached to asparagine using a nanopore is still unfulfilled.^245^

### Prospects of nanopore technologies

Of particular promise are recent demonstrations of enzyme-mediated translocation systems, where engineered molecular motors such as ClpX or Hel308 can pull the same protein molecule through nanopores in a controlled, stepwise manner multiple times.^132^^,^^155^ Meanwhile, the integration of advanced computational methods reinforces this opportunity. Machine learning algorithms, particularly deep learning models trained on extensive datasets of current blockades, are becoming increasingly vital for accurate residue identification and noise reduction. These computational approaches can deal with complex factors such as sequence context effects and transient structural variations that currently challenge interpretation. Furthermore, real-time data processing pipelines will be essential for handling the substantial data streams generated by long-read protein sequencing, potentially enabling immediate functional annotations alongside sequence determination.

As these technical capabilities develop, a promising avenue is clinical diagnostics, particularly due to the unique customizability of nanopores. Besides sequencing, nanopores are able to resolve specific molecules with subtle differences, and therefore it can be a transformative tool in complex samples, including the detections of PTMs, mutations, peptide biomarkers, and PPIs. Currently, the pathogenesis for many well-concerned diseases is still unclear at the molecular level, making the detection of clinical biomarkers an urgent need for early diagnosis and drug development. For example, in AD, the isoform production balance and excessive chemical modification of biomarker proteins in the cerebrospinal fluid of AD patient is expected to be thoroughly analyzed with a solid and safe method.^246^ While traditional techniques, such as immunohistochemistry and ELISA, often suffer from a suboptimal limit of detection (LOD), nanopore offers an alternative. By continuously improving the spatial and temporal sensitivity of nanopores, researchers have achieved site-specific recognition of important PTMs and mutation on the amyloid-β protein.^140^ Moreover, nanopores can detect conformation differences that are undetectable by MS with the same mass-to-charge ratio. With machine learning, identifying enantiomers, observing chiral interconversion, and distinguishing peptides with a single different chiral amino acid are possible.^247^^,^^248^ These features indicate that nanopore-based proteomics has the potential to play an irreplaceable role in clinical problem-oriented research and application.^249^

Nanopore technology is promising for detecting rare peptide biomarkers, particularly in clinical samples, especially when monoclonal antibodies are not available.^250^ Nanopores engineered with aptamers can identify peptide biomarkers without the help of enzymes or antibodies, which avoids non-specific interactions between reagents and untargeted molecules.^251^ Currently, three promising methodologies for quantitative analysis are proposed for future diagnostic platforms. (1) Attach specific probes to the nanopores: Mayse et al. constructed a nanopore sensor equipped with a MLL4_Win_ probe into tFhuA via a flexible Gly/Ser-rich hexapeptide tether, and quantitatively detected WD40 repeat protein 5 (WDR5) through the MLL4win-WDR5 interaction.^252^ (2) Employ “dual-aptamer system”: instead of directly detecting target molecules, the “dual-aptamer system” will release another aptamer as a surrogate reporter after forming a specific binding to increase the capture rate of molecules and improve the accuracy of quantitative detection indirectly. For instance, Rauf et al. measured the cocaine concentration in serum and saliva, which was linearly correlated with the capture rate, reaching a LOD of 50 nM^253^ (3) Bind DNA probes to magnetic beads. Li et al. bound vascular endothelial growth factor (VEGF) to a nucleic acid aptamer, which caused the aptamer-probe duplex to unwind. After incubating VEGF with magnetic beads, the probes were released to produce signature current blockades with signatures used for VEGF quantification.^254^

Beyond traditional protein pores, there appear to be unique nanopores fabricated from other compositions to meet diverse detection requirements. The macrolittin peptide can be used to form a nanopore structure inspired by the bee venom peptide melittin.^255^ Furthermore, researchers have demonstrated the potential of DNA structures as transmembrane information-exchange channels,^256^ such as a triangular DNA nanopore using DNA origami technology.^257^ Although the programmability for precise geometry and size provided by DNA origami nanopores is advantageous, they cannot function spontaneously. Intriguingly, an article published by *Nature* reported the *de novo* design of transmembrane β-barrel (TMβ) nanopore proteins with adjustable sizes and shapes.^258^ Undoubtedly, the non-stop advancement and innovation in related fields will eventually overcome the flaws of traditional nanopores and provides diversity for custom nanopores. At present, nanopore-based point-of-care testing (POCT) platforms for clinical biomarkers are welcome,^259^ and it is believed that nanopore will compete as a supplement to the existing methods, as what nanopore sequencing has done to the first complete, gapless sequence of the human genome.

## Acknowledgments

This work was sponsored by the 10.13039/100014718National Natural Science Foundation of China (No. 32271520) and the 10.13039/100007219Hunan Provincial Natural Science Foundation Enterprise Joint Project (No. 2025JJ90229).

## Author contributions

Y.T. Wang, Q. Han: writing - original draft; Y.L. He and S.M. Deng: literature investigation and visualization; Z.W. Huang and Z.Y. Zhang: format analysis; .J. Dai: supervision, project administration, and writing – review and editing; Z.X. Dong: conceptualization, writing - review and editing, supervision, and project administration.

## Declaration of interests

The authors declare no competing interests.
